# Morphological screens using aged primary adult neuronal, microglial, and astrocytic cultures to find novel neurotherapeutics

**DOI:** 10.3389/fncel.2023.1253192

**Published:** 2023-08-23

**Authors:** Arthur Sefiani

**Affiliations:** ^1^Department of Neuroscience and Experimental Therapeutics, Texas A&M University Health Science Center, Bryan, TX, United States; ^2^NeuroCreis, Inc., College Station, TX, United States

**Keywords:** neurotrauma and neurodegenerative disease, drug screen approaches, neurotherapeutic, primary cell culture, primary adult microglia, primary adult astrocyte, primary adult neuron

## Abstract

The average age of a patient with neurotraumatic injuries or neurodegenerative diseases has been increasing worldwide. The preclinical live animal models used for neurotrauma and neurodegenerative diseases are typically young adults, failing to represent the age of humans in the clinic. This dichotomy in age between human populations and animal models is likely to impede the understanding of the pathological mechanisms of most neurological disorders and the translation of their respective promising therapies. This lack of cohesion between animal models and patients in the clinic begins prior to *in vivo* testing, it starts during the *in vitro* drug screening phase. Conventional screening methods typically involve the use of stem cell derived neural cells, with some researchers using embryonic derived neural cells instead. These cells lack the fundamental characteristics present in aged neural cells, such as age-induced changes in process length and branching in microglia and how astrocytes respond to various insults. Various technologies and techniques have been developed recently that can help researchers use age-appropriate neural cells for their drug discovery endeavors. The use of age-appropriate neural cells during screening phases is hypothesized to significantly increase the translation rate of the hits to the geriatric patients suffering from neurotraumatic and neurodegenerative diseases.

## Introduction

Median age worldwide has been increasing, leading to more senior individuals suffering from age-associated neurological disorders. From 1990 to 2010, total dementia cases almost doubled, and per capita dementia cases increased by 53%. From 1978 to 2005, the percentage of people suffering from a spinal cord injury (SCI) being of geriatric age increased by over 2 1/2-fold (Sefiani et al., 2022). One example of an age-associated neurological disorder is stroke, where 75% of patients are over the age of 65 and patients over 75 years of age are more likely to be hospitalized and have higher mortality rates (Simmons et al., 2023). In the field of stroke, 89% of the preclinical testing is performed in very young stroke animal models while human patients are substantially older. Stroke preclinical studies that use age-appropriate subjects show significantly reduced response to treatment (measured using infarct volume) compared to studies only incorporating young subjects that do not correspond to the human stroke population (McCann and Lawrence, 2020). Therefore, the efficacy of therapeutics cannot be properly assessed in young stroke models if the target patient population is of geriatric age. This dichotomy in age between human populations and animal models is likely to impede the understanding of the pathological mechanisms of most neurological disorders and the translation of their respective promising therapies considering aging itself is the single greatest risk factor for stroke, dementia, and many other neurological diseases (Wahl et al., 2019). Additionally, drugs, such as methylphenidate used to treat attention deficit-hyperactivity disorder, can induce completely opposite effects depending on the patient’s age (Sefiani et al., 2022). Therefore, even in the same species, drugs can have age-dependent effects.

## Neurons

In the process of developing novel therapeutics for neurodegenerative diseases and neurotrauma, the first step is typically to screen in cells while measuring a biologically meaningful parameter to quantify the likelihood of a given candidate to have the intended therapeutic effect. Ideally, the cells should represent the pathological state, such as aged neurons with reduced capacity to regenerate neurites and diminished metabolic activity (Sutherland et al., 2021). Increasing neurite growth and neuron survival in adults has great therapeutic potential. Neurite growth can improve neural communication after neurotrauma and coupled with improved survival, can mitigate necrosis after trauma and maturation of neurodegenerative diseases (Sefiani et al., 2022). Therefore, there is a need for a screening system that recapitulates the regenerative capacity and metabolic activity of aging neurons to develop novel therapeutic strategies for age-associated neurological disorders. Conventional screening methods usually use embryonic or induced pluripotent stem cell (iPSC)-derived neurons that do not recapitulate the lack of therapeutic response seen in adult neurons, which till this day still cannot be reversed, even with genetic manipulation (Geoffroy et al., 2016). Human patient iPSC-derived neurons, which contains the patient’s genetic information, also cannot represent aging neurons; these cells mimic neurons of a developing embryonic brain. This dichotomy in age between *in vitro* assays and *in vivo* settings is likely to play a role in the high number of failures when moving a drug forward along the clinical phases. Therefore, it is vital age-appropriate adult neurons are used in screens that aim to find novel therapeutics. One method of screening in adult neurons is micro-dissecting the cortex of mice of various adult ages. The method involves the removal of debris and blood cells, followed by the separation of neurons from glia using magnetic-activated cell sorting (MACS) technology (Miltenyi Biotec, North Rhine-Westphalia, Germany) to produce a neuron enriched culture. Using this method, researchers illustrated the need for using aged neurons in screens because, even *in vitro*, drugs, such as RO48 and 7-epi Paclitaxel, induce obvious age-dependent effects on neurite growth and survival (Sefiani et al., 2022). Therefore, screening in primary adult neurons provides distinct advantages in finding novel therapeutics for age-associated neurological disorders, hypothesized to more accurately reflect clinical response to a given drug.

## Microglia

Glial function and morphology, impacted by age, are vital components in the progression of neurodegenerative diseases and trauma induced pathology. In healthy central nervous systems (CNS), microglia possess many highly dynamic long, thin, and branched processes that interact with other neural cells, rapidly moving to respond to environmental aberrations. High resting motility of microglia may serve as a maintenance function to clear the CNS of necrotic deteriorated tissue and harmful metabolic products to provide protection for neurons and astrocytes against minor insults (Nimmerjahn et al., 2005). Microglia in the amoeboid morphology do not provide these beneficial attributes, whether it’s because the microglia are either already preoccupied with an insult or the morphology manifested from an underlying pathology (Wendimu and Hooks, 2022). Therefore, there are therapeutic advantages in first, increasing the percentage of microglia in the ramified morphology, then, enhancing the number and length of microglial processes to increase the number of neural cells the microglia can protect by expanding the territory the microglia can positively influence. Aging alters microglial morphology by changing it to a spheroid-activated phenotype reducing processes branching and length, which in turn reduces motility and effectiveness. Aged microglia are in a constantly primed state, becoming hypersensitive to their environment which like an allergic reaction, induces undue inflammatory processes in otherwise benign conditions. These dystrophic microglia produce neurotoxic agents that may make individuals more prone to neurodegenerative diseases. Aging therefore induces a forward feedback cycle, promoting amoeboid morphology which reduces the efficacy of microglia to conduct immune surveillance and protect the CNS from toxic agents which further promotes amoeboid morphology. This process can eventually result in the progression of age-associated neurodegenerative diseases, such as Alzheimer’s and Parkinson’s Disease, which further provoke the amoeboid morphology (Wendimu and Hooks, 2022). Therefore, it is imperative that drug screens are conducted on microglia from aged animals to find compounds that can reverse the priming and hypersensitivity of aged microglia to promote a more ramified morphology that provides neuroprotective characteristics.

Similar to neuronal screening, the use of primary microglia better reflects the *in vivo* microglial activities, including both phenotype and response to insult, compared to immortalized cell lines that merely aim to imitate a few characteristics typically found in microglia. Current microglial isolation methods from primary tissue use shakers to detach microglia from glial populations, resulting in approximately 95% microglial purity. Using this methodology, primary microglia can be isolated from animals of all ages. Larger mammalian brains can be dissected and processed to isolate a greater number of microglia for high-throughput screenings. Previously, screens in microglia aim to identify compounds that produce specific responses, such as inhibiting LPS-induced microglial cell proliferation and proinflammatory cytokine production (Figuera-Losada et al., 2014). New image processing technology has been developed that allows users to quantify microglial morphology, both *in vitro* and *in vivo*, in a high-throughput and unbiased manner (Heindl et al., 2018). Finding compounds that modify microglial morphology toward a more ramified or less active state in adult and diseased brains may reduce microglia’s neuroprotective effects on neurons, therefore, it is essential the culture includes neurons and both the neuronal and microglial morphology are assessed simultaneously (Figuera-Losada et al., 2014). Senescent microglia, which has impaired neuroprotective abilities and has increased secretion of inflammatory molecules (Angelova and Brown, 2019), can also be identified through morphological screens by quantifying the size of the soma. Senescent microglia typically have large and flat morphology (Trias et al., 2019). Altogether, there are many advantages in finding compounds that promote a more ramified state with increased process length and branching, reversing the age and disease induced changes to microglial morphology associated with pathogenesis.

## Astrocytes

Astrocytes play an important role in the regeneration of axons and functional recovery. Astrocyte reactivity does not typically cause an increase in process length, but rather increases ramification and stimulates astrocytic hypertrophy when examined during the acute stages following an insult. Within the second week following an insult, astrocytes demonstrate a less reactive morphology, increasing length of their processes to form a glial scar (Choudhury and Ding, 2016). In the case of SCI, astrocytes can form a glial scar to elongate their processes into bridge like structures that allow for the regeneration of axons across the lesion. Indeed, an increase in the number of GFAP positive processes is correlated with improved hindlimb function (Williams et al., 2015). The therapeutic applications and detrimental effects of reactive astrocytes and glial scarring have been the center of debate for decades. Nonetheless, there is therapeutic potential in increasing the length of astrocytic process to promote axon regeneration and functional recovery after neurotrauma. Predictably, aging also influences astrocytic morphology, reducing the length of processes and increasing process thickness, morphology indicative of a reactive state (Choudhury and Ding, 2016). Astrocytes from aged animals were found to respond to environmental changes differently than astrocytes from young animals. Astrocytes from aged animals had reduced complexity after insult which reduces astrocytic territory, and therefore, reducing ability to support nearby neurons and the plasticity needed to create glial bridges for axon regeneration. The same study found that animals with less complex astrocytes had reduced cognitive abilities, which may be caused by the reduction of metabolic and neuroprotective support astrocytes provide neurons. Indeed, astrocytes from aged animals do have trouble processing neurotoxins thought to be the result of reduced metabolic activity (Palmer and Ousman, 2018). Aging alters both astrocytic morphology and response to biologically active compounds, therefore, it is imperative that adult astrocytes are used in screens aimed to find novel compounds that enhance astrocytic morphology for therapeutic purposes.

To the best of our knowledge, no screens have been developed that aim to quantify the morphological changes of astrocytes *in vitro*. Notably, astrocytes have a more reactive phenotype *in vitro* compared to *in vivo* which can perturb the accurate assessment of compounds on astrocyte morphology. Researchers have developed hydrogel based 3D cultures that allow for astrocyte growth in a stellate morphology to more accurately resemble *in vivo* astrocyte morphology (Gottipati et al., 2020). Another group developed a protocol for coculturing astrocytes with microglia and neurons to more accurately portray the neuroinflammatory profile and response seen *in vivo* (Goshi et al., 2020). Neither method has been used thus far to assess morphological changes in astrocytes. Therefore, there is a need to develop an assay that accurately assesses changes in astrocytic morphology. It is important the assay preserves an accurate astrocytic response to inflammatory insults to better represent the inflammatory induced morphological changes observed *in vivo*. Screening for senescent astrocytes is also important, especially since the prevalence of senescent astrocytes increases with age which may contribute to pathogenesis. Senescent astrocytes can be detected using morphological screens by identifying cells with flattened and enlarged soma (Cohen and Torres, 2019). Altogether, measuring morphological changes in primary aged astrocytes represents a novel approach in identifying compounds that increase astrocytic functionality for neurotherapeutic purposes.

## Discussion

Currently, there are no FDA-approved treatments available for promoting neurological recovery following many age-associated neurological disorders, including neurotrauma, stroke, and most neurodegenerative diseases. It is hypothesized that this is partially due to the lack of age-appropriate preclinical models that accurately resemble the human population in the clinic. Lack of attention to aging as a biological variable indeed starts earlier on, during the screening phases of drug discovery. Screenings are typically done on iPSC-derived or embryonic neural cells that lack fundamental characteristics, such as age-induced epigenetic changes, that is believed to be at least partially responsible for the provocation of age-associated neurological disorders. These epigenetic changes may include morphological changes or reduction in regenerative capacity of neural cells. Compounds that are found effective in younger cells may not target the correct pathways to induce recovery in the aging neural populations found in the target patient population. Therefore, there is a clear need to develop primary adult neuronal, microglial, and astrocytic morphological assays for drug screening. It is important to note, morphological assays may only provide a first indication of efficacy, soliciting further assessment of the compound. Assessing efficacy of a compound in different types of assays, such as functional and molecular assays, would strengthen its justification for use in therapeutics. Examples of functional and molecular assays include measuring how well primary adult glia can phagocytose debris and produce protective cytokines. For astrocytes specifically, one could measure the speed at which astrocytes migrate to insults and the cytokines released in the process.

One disadvantage of using primary adult neural cells is the invasive procedure required to collect enough cells to conduct the screenings in, resulting in the death of the subject the cells are collected from. Therefore, primary neural cells must be collected from non-human animals. This may pose as an issue due to genetic differences between species and certain neurological conditions not occurring in rodents, such as wild-type mice not developing neurofibrillary tangles at any age (Yokoyama et al., 2022). There are also many morphological and physiological differences between human and rodent neurons, such as neurite length and resting potential (Mihaljević et al., 2021). Regardless, drug screens assess relative change, therefore, absolute differences between species may not be an inherent issue. The use of human patient-derived cells can be used in screenings for genetic neurological disorders, supplying newborn neurons carrying the patient’s genetic information. Though, its ability to recapitulate the diseased state adult neural cells are in during pathogenesis decades after birth is greatly reduced, as patient-derived cells are still newborn cells lacking the epigenetic changes that occur with aging (Slanzi et al., 2020). Lack of incorporation of the age-factor in screenings is hypothesized to be a greater contributing factor to failures in the clinic than the genetic factor. Possible workarounds to the genetic issue are to use primary cells from animals that better resemble human aging and pathologies, such as non-human primates, followed by co-screening in human iPSC-derived cells as a secondary vetting measure. Altogether, the use of primary adult cells is hypothesized to increase the clinical relevance of the findings and expedite the translation of bench side research into the clinic.

## Data availability statement

The original contributions presented in this study are included in the article/supplementary material, further inquiries can be directed to the corresponding author.

## Author contributions

AS conceptualized the ideas and wrote the manuscript.
